# GALENICALS IN THE TREATMENT OF CRUSTED SCABIES

**DOI:** 10.4103/0019-5154.62748

**Published:** 2010

**Authors:** P Sugathan, Abhay Mani Martin

**Affiliations:** *From the Division of Dermatology, Baby Memorial Hospital, Indira Gandhi Road, Calicut – 673 004, India.*

**Keywords:** *Crusted scabies*, *galenicals*

## Abstract

Crusted scabies is rare. It is a therapeutic challenge, as the common drugs used against scabies are unsatisfactory. The successful use of galenicals in a 10-year-old girl with crusted scabies is reported.

## Introduction

Crusted scabies is rarely encountered in clinical practice. It has re-emerged recently with increasing number of immunosuppressed[1] patients: diabetics, transplant recipients, AIDS patients, and patients on chronic immunosuppressive drugs. Medications used in the treatment of scabies are given in Table 1. These therapies have shown excellent therapeutic and safety profile. Ivermectin given orally has recently gained wider acceptance.[2] However, the efficacy of these drugs is unsatisfactory in crusted scabies, as they fail to penetrate the thick adherent scales and crusts.

**Table 1:** Therapeutic options in the treatment of scabies

Ung. sulfuris 10%	Benzyl benzoate emulsion 25%	Tetmasol 20% tincture
Mesulphan	Gamma benzene hexachloride	Permethrin 5% cream
Crotamiton 10% cream	Ivermectin oral 200 μg/kg	Australian tea tree oil

Galenicals were once the mainstay of therapy in dermatology. This has gone out of favor lately because of the lack of expertize in preparing these medications and the availability of more convenient and cosmetically elegant preparation.

An unusual case of crusted scabies which was effectively treated with galenicals is described below.

## Case Report

**Figure 1:**
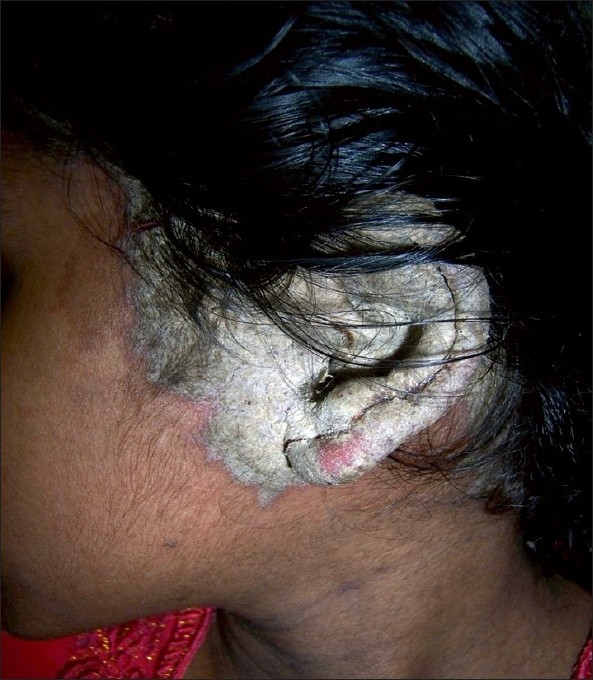
Extreme hyperkeratosis of the left pinna

**Figure 2:**
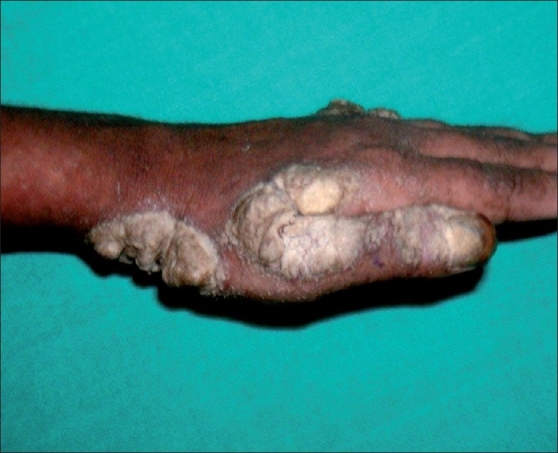
Shows extreme hyperkeratosis and adherent crusts on the wrist and first interdigital space

A 10-year-old girl was brought by her parents with complaints of thick, adherent, and scaling lesions of 2 year duration involving the elbows, wrists, ears, scalp, and knees [Figures 1 and 2]. She had consulted a dermatologist previously who made a provisional diagnosis of psoriasis and treated her with topical steroids and salicylic acid. The lesions did not subside with this. She had in addition weight loss and a chronic productive cough which was diagnosed as pulmonary tuberculosis.

**Figure 3:**
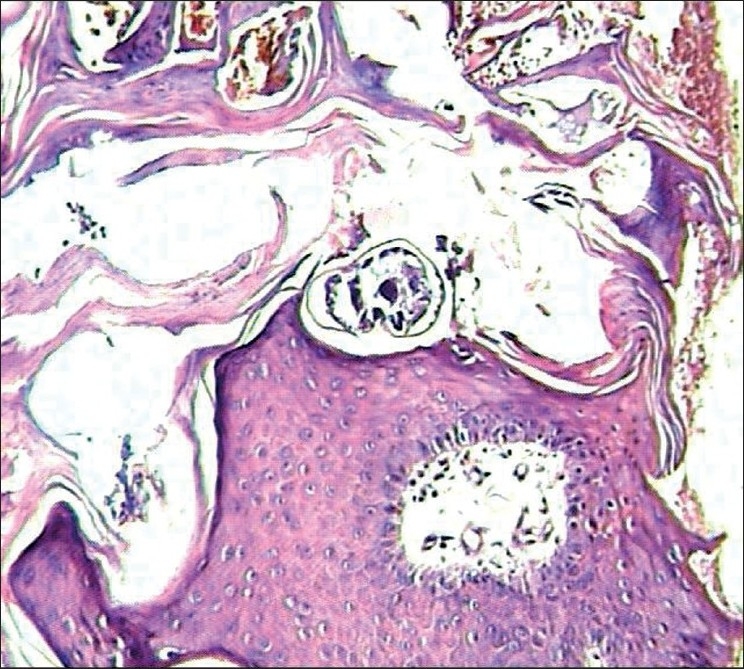
Biopsy from the elbow showing acanthosis hyperkeratosis and several burrows showing mites. (H & E Stain, ×40)

A diagnosis of crusted scabies was made based on the clinical picture. Scrapings from the thick crusts showed numerous scabies mites on KOH mount. A skin biopsy from the lesion showed irregular acanthosis, hyperkeratosis, and several mites in their burrows in the stratum coneum [Figure 3].

**Figure 4:**
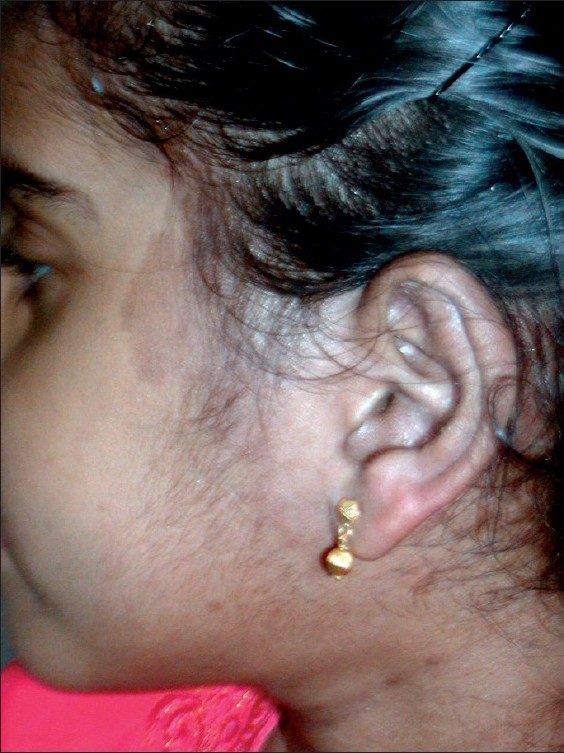
Three days after treatment with galenical ointment shows clearance of all lesions

She was treated initially with Tab. Ivermectin 200 μg/kg and then with topical gamma benzene hexachloride with no relief. She was then treated with a galenical preparation comprising salicylic acid 3%, unguentum hydrargyrum ammoniatum dilutum (Ung HAD) 5%, liquor picis carbonis 7%, purified white vaseline to make 100%, to apply on the affected areas twice daily. There was prompt relief of signs and symptoms and she reported back 2 weeks later with total disappearance of all lesions [Figure 4].

## Discussion

Crusted scabies is an unusual variant of scabies which is a diagnostic and therapeutic challenge for the treating dermatologist. The causative agent *Sarcoptes scabiei var hominis* shows uncontrolled proliferation due to inability of the individual to mount an immune response to the mite. Scrapings and skin biopsy from the lesion show mites teeming through the slide.

Treatment options in crusted scabies are limited and are not satisfactory. Most topical preparations like permethrin, benzyl benzoate, and gamma benzene hexachloride or ivermectin which are used for classical scabies do not produce predictable results as they fail to penetrate the thick and adherent scales and crusts.[3] The active ingredient in this preparation is ammoniated mercury which is a white crystalline powder, which when incorporated in ointments, selectively poisons the acarus scabiei. Two types of mercury ointments were available in the past. One called Unguentum Hydrargyrum had 33% mercury, which was used to treat bacterial infections, was too toxic after absorption through the skin, and is not available. The dilute ammoniated mercury (5%) is safe and effective in the treatment of crusted scabies as well as one of the ingredients in an ointment used regularly to treat psoriasis.[4] Clinical manifestation of chronic mercury poisoning such as stomatitis, erythrism, tremors, and acrodynia develop only on prolonged use and has never been reported from local application. It has no systemic effects at all on short-term use. Salicylic acid is a keratolytic agent. It loosens scales by breaking the disulfide bridges between the corneocytes. Alcoholic solution of coal tar is a keratoplastic. Galenicals, as a form of treatment in dermatology, require expertise to prepare the medications. A strong pharmacy back up is essential to prepare medications of good quality. The medicines are effective if used judiciously and is affordable to the average Indian patient.

## Conclusion

The above case illustrates the effective use of galenical preparations in the therapy of crusted scabies that failed to respond to routinely used therapeutic measures. This case reiterates the fact that galenical preparations still hold much potential in spite of rich claims made by fixed dose preparations available in the market. The greatest advantage of these galenical preparations is that they can be titrated and tailor-made to suit the patient needs by varying the concentrations of the individual constituents.
